# Unraveling the enigma of sarcopenia and sarcopenic obesity in Indian adults with type 2 diabetes – a comparative cross-sectional study

**DOI:** 10.1186/s40842-024-00179-4

**Published:** 2024-06-17

**Authors:** Yogesh M., Monika G. Patel, Hardik Harshadbhai Makwana, Hardikkumar Kalariya

**Affiliations:** 1 Department of Community Medicine, M P Shah Government Medical College, New PG Hostel, Shri MP Shah Medical College campus, GG Hospital, Patel Colony Post, Jamnagar, Gujarat 361008 India; 2 Department of Pathology, GMERS Medical College, Junagadh, Gujarat India

**Keywords:** Sarcopenia, Diabetes, India, Prevalence, Complication

## Abstract

**Background:**

Sarcopenia and sarcopenic obesity are growing concerns associated with increasing diabetes incidence, but data from Indian diabetic cohorts are limited. This study examined the prevalence and clinical factors associated with sarcopenia and sarcopenic obesity.

**Methods:**

In this cross-sectional study, 750 participants aged 35–70 years were recruited by systematic stratification and a fixed quota sampling technique from medical camps and categorized into diabetic (*n* = 250), nondiabetic (*n* = 250), and obese nondiabetic (*n* = 250) groups. The assessments included questionnaires, muscle mass estimation by bioimpedance analysis, and blood tests. Sarcopenia was defined using the Asian Working Group consensus, and sarcopenic obesity was defined as sarcopenia with a BMI ≥ 25 kg/m2. Logistic regression was used to analyze risk factors.

**Results:**

Sarcopenia affected 60% of diabetic patients, 28% of nondiabetic patients, and 38% of nonobese nondiabetic patients (*p* < 0.001). The prevalence of sarcopenic obesity was 40%, 11%, and 30%, respectively (*p* < 0.001). Diabetes was associated with 2.3-fold greater odds (95% CI 1.1–4.7) of sarcopenia and 2.4-fold greater odds (1.1-5.0) of sarcopenic obesity after adjustment. A duration greater than 10 years, uncontrolled diabetes, age greater than 65 years, low physical activity, hypertension, and dyslipidemia also independently increased the odds.

**Conclusion:**

Indian adults with type 2 diabetes have a high burden of sarcopenia and sarcopenic obesity. Early optimization of diabetes care and lifestyle changes are vital for preserving muscle health.

## Introduction

Sarcopenia is characterized by a progressive decline in muscle mass, strength, and physical performance, leading to functional limitations, frailty, and an increased risk of adverse health outcomes, such as falls, disability, and mortality. In individuals with T2DM, sarcopenia may exacerbate existing complications, including impaired glucose homeostasis, cardiovascular disease, and reduced quality of life [1, 2]. The revised European Consensus on the Definition and Diagnosis of Sarcopenia (EWGSOP2) provides a comprehensive framework for identifying and assessing sarcopenia, incorporating measures of muscle strength, quantity or quality, and physical performance [3].

A systematic analysis of sarcopenia incidence revealed significant heterogeneity across studies. The reported prevalence ranged from 10 to 27%, with variations attributable to the diagnostic criteria employed. Notably, the highest and lowest prevalence rates were observed in Oceania and Europe, respectively, when using the EWGSOP and EWGSOP2 classifications. Furthermore, the analysis indicated an influence of age, with prevalence estimates between 8% and 36% in younger individuals (< 60 years) and 10–27% in those aged 60 and above. Interestingly, gender disparities emerged based on the chosen criteria. Men exhibited a greater prevalence of sarcopenia according to the EWGSOP2 (11% vs. 2%), while the International Working Group on Sarcopenia definition showed a greater prevalence of sarcopenia in women (17% vs. 12%). Finally, the prevalence of severe sarcopenia ranged from 2 to 9%. These findings highlight the need for standardized diagnostic criteria and the potential influence of age and sex on sarcopenia incidence [4].

Type 2 diabetes mellitus (T2DM) and sarcopenia create a vicious cycle that can significantly worsen health outcomes for patients. The problems were muscle loss worsens diabetes; sarcopenia, characterized by progressive muscle mass and functional decline, can exacerbate T2DM complications; impaired glucose control, reduced muscle mass leads to decreased insulin sensitivity and glucose uptake, hindering blood sugar control, a hallmark of T2DM [5]; and cardiovascular risk, both T2DM and sarcopenia are independent risk factors for CVD. The combination further elevates CVD risk due to factors such as impaired blood flow and endothelial dysfunction [6] and decreased quality of life: reduced mobility and functional limitations associated with sarcopenia negatively impact daily activities and overall well-being in T2DM patients [7].

The European Working Group on Sarcopenia in Older People (EWGSOP2) recognized the importance of early intervention [3]. Routine screening and assessment of sarcopenia in high-risk populations, including those with T2DM, are crucial for mitigating these negative consequences.

The emergence of sarcopenic obesity presents a significant challenge due to the coexistence of sarcopenia and excessive adiposity. This complex condition, characterized by the combination of muscle wasting and obesity, poses unique difficulties because it can exacerbate functional decline and increase the risk of associated complications. The interplay between these two conditions contributes to a dual burden that impacts physical health and overall well-being, emphasizing the need for targeted interventions to address the specific challenges posed by sarcopenic obesity [8]. A recent analysis revealed a wide range of prevalence rates for sarcopenic obesity among elderly populations, ranging from 4.4% to a striking 94%, depending on the diagnostic criteria employed [9]. Although the data are limited, studies suggest that SO might be a concern in India. A study involving elderly adults in India reported a prevalence of SO between 5.4% and 6.3% using different diagnostic criteria [10]. This variability highlights the complex nature of this condition and the need for comprehensive approaches to address it effectively.

In the context of Asia, where the prevalence of both sarcopenia and obesity is on the rise, several studies have pointed to a concerning trend: a significant co-occurrence of sarcopenia and obesity within populations with type 2 diabetes mellitus (T2DM) [10–14]. This correlation underscores the intricate interplay between metabolic disorders and musculoskeletal health, further emphasizing the urgency of understanding and addressing these issues within diverse population groups.

The mechanisms underlying the development of sarcopenia are multifactorial and involve a complex interplay of factors such as sedentary lifestyles, nutritional deficiencies, chronic inflammation, and insulin resistance [15–17]. Diabetes and obesity, both highly prevalent conditions globally, have been implicated in accelerating the loss of muscle mass and function. In diabetes, factors such as hyperglycemia, the accumulation of advanced glycation end products, and mitochondrial dysfunction contribute to muscle wasting, exacerbating the progression of sarcopenia [18]. Similarly, obesity fuels inflammatory pathways and exacerbates vitamin D deficiencies, compromising muscle quality and physical function [19–21].

Despite the growing body of research on sarcopenia and sarcopenic obesity, there remains a notable gap in our understanding, particularly regarding their prevalence and associated risk factors within diabetic Asian cohorts. This study seeks to address this gap by investigating the prevalence of sarcopenia and sarcopenic obesity, alongside their clinical correlates, within diabetic, nondiabetic, and obese nondiabetic groups in Gujarat, India. By shedding light on these issues, we aim to pave the way for more targeted interventions and improved management strategies tailored to the unique needs of diverse populations.

## Methodology

### Study design and sample

This cross-sectional study utilized systematic sampling with stratification and fixed quota sampling to recruit 750 participants from diabetes and medical camps in Gujarat, India, between January 2023 and January 2024. Based on the inclusion and exclusion criteria, 250 diabetic patients, 250 nondiabetic healthy controls, and 250 nondiabetic obese adults aged 35–70 years were enrolled. The sample size was calculated based on a confidence level of 95% and a margin of error of 5%.

The sample size was determined based on previous recommendations and the literature on sarcopenia incidence. Studies suggest that sample sizes of at least 100–150 participants per group provide sufficient power in sarcopenia-related research [22, 23].

Specifically, Morley et al. [23]. noted sample sizes ranging from 100 to over 400 have been commonly used in cross-sectional studies of sarcopenia depending on the primary outcome measurement. Beaudart et al. [22] recommended ≥ 100 subjects per group for studies examining muscle mass parameters.

Therefore, with our projected sample size of 250 participants in each of the diabetes, nondiabetes, and obese nondiabetes groups, we expected to have adequate statistical power (> 80%) to detect differences in sarcopenia and related variables among the three groups. Comparing equal group sizes also allows for the detection of smaller effect sizes in parameters across groups. The sample provides adequate numbers to conduct bivariate and multivariate analyses to assess for independent associations between sarcopenia and sarcopenic obesity.

### Inclusion criteria

- Diagnosed type 2 diabetes patients aged 35–70 years.
- Nondiabetic healthy adults aged 35–70 years.
- Nondiabetic obese adults aged 35–70 years with a BMI ≥ 25 kg/m2.
- Provided informed consent.

### Exclusion criteria

- Type 1 diabetes.
- Taking medications that affect muscle mass (e.g., steroids, thyroid hormone therapy).
- Diagnosis of cancer, kidney disease, liver disease, or thyroid disease.
- Bedridden or wheelchair-bound patients.
- Unable to undergo body composition analysis or physical assessments.

### Sampling technique

**Fig. 1:**
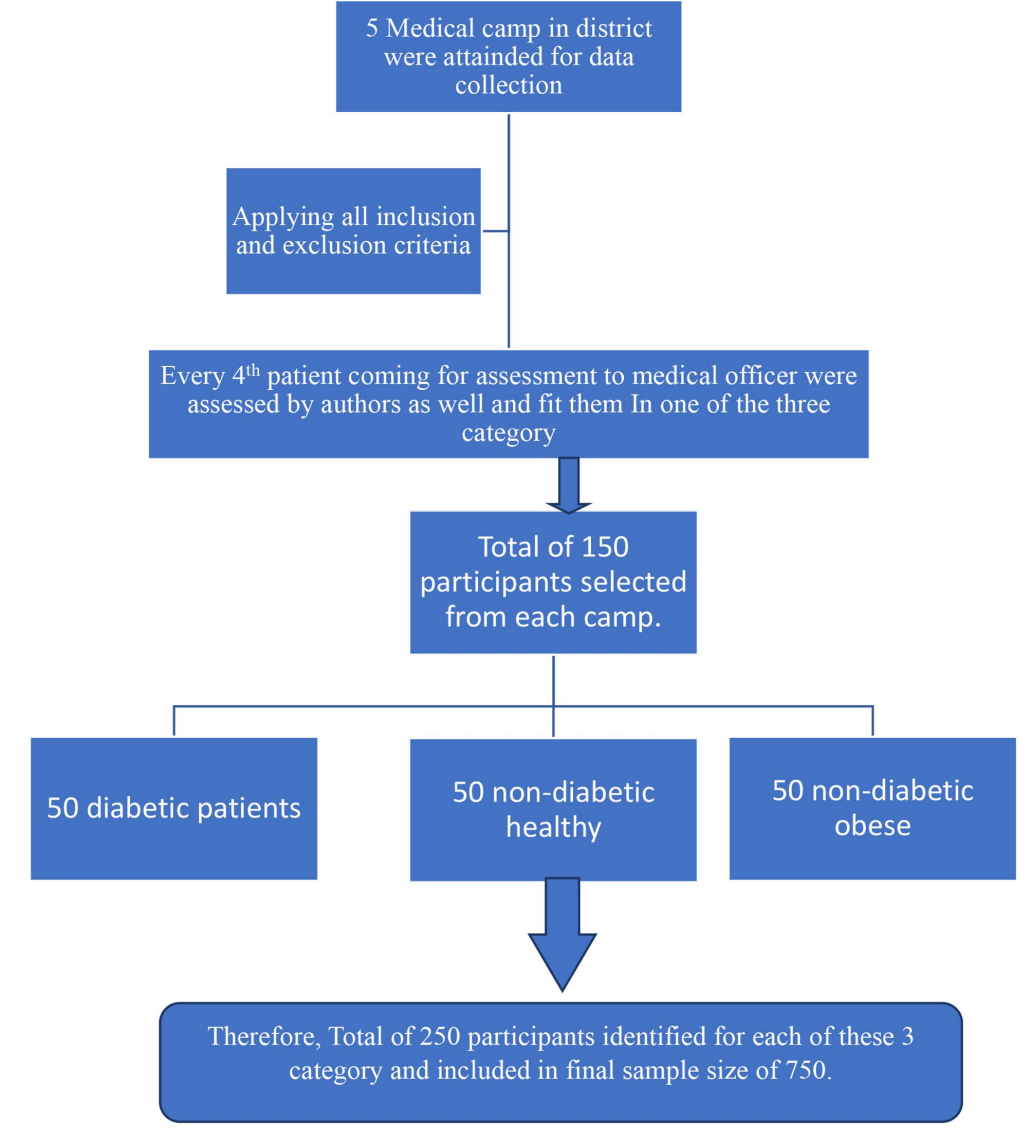
Participant recruitment procedure

This study employed a systematic sampling approach with stratification and fixed quota sampling, facilitating the enrollment of diabetic patients alongside matched nondiabetic obese/healthy controls from the same population. Participants were systematically selected, every 4th patient, from each medical camp, ensuring representation across predefined categories: diabetic patients, nondiabetic healthy controls, and nondiabetic obese adults aged 35–70 years. With a fixed quota of 50 participants for each category, sampling continued until completion, ensuring a balanced and representative sample from each group while maintaining systematic selection across all medical camps. (Fig. 1)

### Data collection

Data collection was carried out by a trained team of investigators using a structured questionnaire and medical examinations. The questionnaire collected demographic information, medical history, physical activity levels using the Global Physical Activity Questionnaire (GPAQ) [24], and duration of diabetes for those participants. Physical examinations included blood pressure, height, weight, and fasting blood sample collection. The process of obtaining informed consent adhered to the principles outlined in the Declaration of Helsinki and good clinical practice guidelines.

Prior to recruitment, all potential participants were provided with a detailed information sheet in their local vernacular language. This information sheet outlined the purpose of the study, the procedures involved, the potential risks and benefits, the voluntary nature of participation, and the measures taken to ensure confidentiality and data protection. The study investigators or trained research staff then engaged in a comprehensive discussion with each potential participant, ensuring that they had a clear understanding of the study details and addressing any queries or concerns they might have had. Participants were explicitly informed of their right to withdraw from the study at any point without any consequences.

For those willing to participate, written informed consent was obtained by having them sign or thumb-print a consent form in the presence of a witness. In cases where participants were unable to provide written consent due to literacy or physical limitations, verbal consent was obtained and documented by a witness who attested to the participant’s voluntary agreement.

### Assessment of body composition and definitions

Body mass index (BMI) was calculated as weight (kg)/height (m)2. Participants underwent bioimpedance analysis (BIA) using a Tanita body composition analyzer to estimate muscle mass [25]. Appendicular skeletal muscle mass (ASM) was calculated as the sum of muscle mass in all four limbs. Sarcopenia was defined using the Asian Working Group for Sarcopenia criteria of low muscle mass + low muscle strength and/or physical performance [26]. Sarcopenic obesity was defined as sarcopenia + obesity (BMI ≥ 25 kg/m2) [8].

### Laboratory investigations

Overnight fasting blood samples were assessed for glycated hemoglobin (HbA1c) and serum lipids, including total cholesterol (TC), triglycerides (TG), low-density lipoprotein cholesterol (LDL-C), and high-density lipoprotein cholesterol (HDL-C) [27].

### Statistical analysis

The statistical analysis for this study was performed using SPSS version 26.0 (IBM Corp., Armonk, NY, USA). Descriptive statistics were computed to summarize the characteristics of the study population. Continuous variables were presented as mean ± standard deviation, while categorical variables were expressed as frequencies and percentages. Between-group comparisons for continuous variables were conducted using one-way analysis of variance (ANOVA) followed by post hoc analysis using Tukey’s honest significant difference (HSD) test or the Games-Howell test, depending on the homogeneity of variances. The homogeneity of variances was assessed using Levene’s test. For categorical variables, between-group comparisons were performed using the chi-square test or Fisher’s exact test, as appropriate. The prevalence of sarcopenia and sarcopenic obesity in each group (diabetic, non-diabetic, and obese non-diabetic) was calculated and compared using the chi-square test or Fisher’s exact test. To investigate the associations between potential risk factors and the outcomes of sarcopenia and sarcopenic obesity, bivariate and multivariate logistic regression analyses were performed. Crude odds ratios (ORs) and their corresponding 95% confidence intervals (CIs) were calculated in the bivariate analysis. Variables found to be statistically significant in the bivariate analysis, along with clinically relevant variables, were included in the multivariate logistic regression models to estimate adjusted odds ratios (AORs) and 95% CIs.

Multicollinearity among independent variables was assessed using the variance inflation factor (VIF), and variables with a VIF greater than 5 were considered to have collinearity issues and were excluded from the multivariate models. The assumptions of logistic regression, including linearity of the logit and absence of influential observations, were evaluated using appropriate diagnostic plots and statistical tests. For all statistical analyses, a two-tailed *p*-value less than 0.05 was considered statistically significant.

## Results

The prevalence of sarcopenia and sarcopenic obesity is notably elevated among individuals with diabetes compared to both nondiabetic and obese nondiabetic individuals. Table 1 shows a significantly greater prevalence of sarcopenia in the diabetes group (60% or 151 out of 250 subjects) than in the nondiabetes group (28% or 71 out of 250 subjects) and obese nondiabetes group (38% or 96 out of 250 subjects). The prevalence of sarcopenic obesity was also significantly greater in the diabetes group (40% or 99 out of 250 subjects) than in the nondiabetic (11% or 27 out of 250 subjects) and nonobese (30% or 76 out of 250 subjects) groups (*p* < 0.001).

**Table 1:** Prevalence of sarcopenia and sarcopenic obesity in different groups

Group	No. of subjects	No. with sarcopenia (%)	No. with sarcopenic obesity (%)	*P* value
Diabetes	250	151 (60.4%)	99 (39.6%)	< 0.001 **
Nondiabetes	250	71 (28%)	27 (11%)	< 0.001 **
Obese nondiabetes	250	96 (38%)	76 (30%)	< 0.001 **

*P < 0.05, *-significant; **P < 0.001, highly significant

Table 2 outlines several clinical differences between the groups. The diabetes group had a lower mean BMI of 24.5 ± 3.2 kg/m2 compared to 23.1 ± 2.8 kg/m2 in the nondiabetic group and 32.6 ± 4.1 kg/m2 in the nonobese group. HbA1c was greater in the diabetes group (7.9 ± 1.1% versus 5.6 ± 0.3% (nondiabetes) versus 5.5 ± 0.4% (obese nondiabetic)). The diabetes group also had lower mean TC (172 ± 35 mg/dL), LDL-C (92 ± 27 mg/dL), and HDL-C (47 ± 11 mg/dL) but greater TG (156 ± 67 mg/dL) than the other two groups.

**Table 2:** Clinical Characteristics of the Study Groups

Characteristic	Diabetes	Nondiabetes	Obese nondiabetes	*P* value
Age (years)	65.2 ± 5.8	63.5 ± 6.1	64.8 ± 5.9	0.34
Gender (M/F)	151/99	126/124	134/116	0.45
BMI (kg/m2)	24.5 ± 3.2	23.1 ± 2.8	32.6 ± 4.1	< 0.001 **
HbA1c (%)	7.9 ± 1.1	5.6 ± 0.3	5.5 ± 0.4	< 0.001 **
SBP (mmHg)	134 ± 19	128 ± 14	132 ± 18	0.23
DBP (mmHg)	76 ± 10	74 ± 8	78 ± 12	0.13
TC (mg/dL)	172 ± 35	187 ± 33	197 ± 37	0.004 *
LDL-C (mg/dL)	92 ± 27	114 ± 29	121 ± 31	< 0.001 **
HDL-C (mg/dL)	47 ± 11	53 ± 12	42 ± 9	< 0.001 **
TG (mg/dL)	156 ± 67	139 ± 54	189 ± 76	0.001 **

*P < 0.05, *-significant; **P < 0.001, highly significant

Table 3 shows that diabetes had an adjusted odds ratio (AOR) of 2.3 (95% CI 1.1–4.7) for associations with sarcopenia. Diabetes duration ≥ 10 years, uncontrolled diabetes (HbA1c ≥ 8%), age ≥ 65 years, low physical activity, hypertension, and dyslipidemia were also associated with increased AORs.

**Table 3:** Bivariate and multivariate analyses of factors associated with sarcopenia

Variable	COR (95% CI)	AOR (95% CI)
Diabetes		
- Yes	3.0 (1.5–6.1) *	2.3 (1.1–4.7) *
- No	1	1
Diabetes duration		
- ≥10 years	3.5 (1.0-6.2) *	4.1 (2.9–8.9) *
- <10 years	1	
Uncontrolled diabetes		
- Yes (HbA1c ≥ 8%)	2.1 (1.9–7.2) *	2.8 (1.2–6.1) *
- No	1	1
Obesity		
- Yes	1.8 (0.9–3.4)	1.6 (0.8–3.2)
- No	1	1
Age		
- ≥65 years	2.6 (1.3–5.2) *	2.3 (1.2–4.5) *
- <65 years	1	1
Physical activity		
- Low	3.2 (1.4–7.1) *	2.8 (1.3–6.2) *
- Moderate	1.1 (0.5–2.5)	1.0 (0.4–2.3)
- High	1	1
Hypertension		
Yes	2.4 (1.7–4.7) *	2.3 (1.5–3.8) *
No	1	1
Dyslipidemia		
yes	2.8 (1.9–3.6) *	2.5 (1.8–3.8) *
No		

*P < 0.05, *-significant; **P < 0.001, highly significant

Similarly, in Table 4, diabetes had an AOR of 2.4 (95% CI 1.1-5.0) for associations with sarcopenic obesity. Moreover, longer diabetes duration, uncontrolled diabetes, obesity, older age, low physical activity, hypertension, and dyslipidemia were associated with greater AORs.

These findings underscore the intricate interplay between diabetes and musculoskeletal health, shedding light on potential avenues for targeted intervention and management strategies.

**Table 4:** Bivariate and multivariate analyses of factors associated with sarcopenic obesity

Variable	COR (95% CI)	AOR (95% CI)
Diabetes		
- Yes	2.7 (1.3–5.4) *	2.4 (1.1-5.0) *
- No	1	1
Diabetes duration		
- ≥10 years	2.3 (1.0-5.5) *	2.0 (1.9–4.5) *
- <10 years		
Uncontrolled diabetes		
- Yes (HbA1c ≥ 8%)	2.3 (1.9–5.9) *	2.6 (1.8–5.2) *
- No	1	1
Obesity		
- Yes	3.3 (1.5–7.2) *	2.9 (1.4–6.1) *
- No	1	1
Age		
- ≥65 years	1.8 (0.9–3.7) *	1.6 (0.8–3.2) *
- <65 years	1	1
Physical activity		
- Low	2.5 (1.1–5.8) *	2.1 (1.0-4.7) *
- Moderate	0.7 (0.3–1.7)	0.6 (0.3–1.4)
- High	1	1
Hypertension		
Yes	2.1 (1.6–3.7) *	1.89 (1.4–2.8) *
No	1	1
Dyslipidemia		
yes	3.2 (1.1–6.6) **	3.5 (2.8–6.8) **
No	1	1

*P < 0.05, *-significant; **P < 0.001, highly significant

## Discussion

This cross-sectional study revealed a greater burden of sarcopenia (60%) and sarcopenic obesity (40%) in the diabetic group than in the nondiabetic group. These rates were significantly higher than those in prior research in Korea [28], which reported that the prevalence of sarcopenia ranged from 4.1 to 10.9% in elderly diabetic adults according to different diagnostic criteria. As we selected participants from the camp, due to Berkson’s bias, our population had more severe diabetes, which contributed to the very high prevalence of diabetes.

Similarly, a Chinese study by Chen et al. (2019) [26] utilized the same Asian Working Group diagnostic criteria for sarcopenia and reported an 18.6% prevalence of sarcopenia in their diabetic population. However, the mean BMI was greater in our cohort (24.5 kg/m2 vs. 21.7 kg/m2), possibly indicating more muscle loss. This finding can be compared with that of a previous study [29] that revealed a negative association between BMI and sarcopenia. This means that people with a lower BMI tend to have a greater risk of sarcopenia. This finding aligns with the idea that a lower BMI may indicate less overall muscle mass.

The sarcopenic obesity rate in our diabetic group aligns closely with that reported in a prior Indian study [30], which reported a 30% prevalence of sarcopenic obesity in diabetic patients. In terms of risk factors, our logistic regression analyses revealed many established associations between sarcopenia and sarcopenic obesity. Age over 65 years had 2- to 3-fold increased odds for both conditions, consistent with well-evidenced risks tied to aging [30–32], diabetes duration over 10 years, and uncontrolled diabetes (HbA1c over 8%), which were also independently associated with increased odds of sarcopenia and sarcopenic obesity in the present study. Multiple studies corroborate diabetes and poor glycemic control as predictors of accelerated muscle deterioration and functional decline [30, 33, 34].

Low physical activity levels also greatly increased odds, aligning with the consensus that sedentarism promotes loss of muscle mass and quality [35]. Hypertension and dyslipidemia also emerged as factors potentially indicative of compounding cardiovascular and metabolic strain impacting muscular health. Similarly, previous studies [36–42] also revealed that hypertension and dyslipidemia are related to sarcopenia and sarcopenic obesity [43–45].

This study provides uniquely high yet plausible estimates of the prevalence of sarcopenia and sarcopenic obesity in Indian diabetic patients and of sex variation in the prevalence of these conditions [46]. This study also provides further evidence that diabetes (and its duration and control) is a pivotal contributor to muscle deterioration, especially when it is coupled with aging or obesity. Early screening and lifestyle interventions remain vital for maintaining strength and function in those with diabetes.

### Limitations

This study has several limitations that warrant consideration. Firstly, its cross-sectional design precludes the establishment of causal relationships between the identified risk factors and the development of sarcopenia or sarcopenic obesity. Longitudinal studies are necessary to elucidate the temporal relationship and potential bidirectional interplay between these conditions and the associated factors. Additionally, the reliance on bioimpedance analysis (BIA) for muscle mass estimation, instead of more robust techniques like dual-energy X-ray absorptiometry (DXA), may have led to some misclassification of cases. The high prevalence of sarcopenia and sarcopenic obesity observed in this study could also suggest that the exclusion criteria may have inadvertently missed certain comorbidities or medications that could influence muscle function and mass.

To address these limitations, future research should consider conducting community-based longitudinal studies with rigorous diagnostic methods and comprehensive assessments of potential confounding factors. Such studies would provide valuable insights into the incidence and progression of sarcopenia and sarcopenic obesity, particularly in the context of diabetes onset and progression. Furthermore, interventional studies exploring targeted strategies, such as exercise regimens, nutritional interventions, or adjunctive pharmacotherapies, are warranted to develop effective prevention and management approaches tailored to the unique needs of sarcopenic diabetic patients in the Indian population.

### Recommendations

The high prevalence of both pathologies in diabetic patients in India needs further study. A community study with longitudinal follow-up, especially after diabetes or obesity onset, would better elucidate the temporal relationship between risk factors and muscle deterioration. Tests of targeted interventions such as exercise or nutritional regimens are also needed for sarcopenic diabetic patients in this region. Although consensus diagnostic criteria and cutoff values for sarcopenic obesity are still evolving, our study shows that diabetes management for sarcopenia prevention is vital. Future studies should explore whether pharmacotherapies assist lifestyle change efforts.

## Conclusion

This study has shed light on the alarmingly high prevalence of sarcopenia and sarcopenic obesity among Indian adults with type 2 diabetes mellitus. The findings underscore the urgent need for early recognition and targeted interventions to mitigate the detrimental impacts of these conditions on physical function, quality of life, and overall health outcomes. By identifying modifiable risk factors such as diabetes duration, glycemic control, hypertension, dyslipidemia, and physical inactivity, this study provides a roadmap for preventive and therapeutic strategies. As the global burden of diabetes and age-related muscle deterioration continues to escalate, concerted efforts from healthcare professionals, policymakers, and public health authorities are imperative. Prioritizing muscle health preservation through early diabetes management, lifestyle modifications, and tailored interventions could potentially alleviate the mounting societal and economic costs associated with sarcopenia and sarcopenic obesity. By addressing this intricate interplay between metabolic disorders and musculoskeletal health, we can pave the way towards healthier aging and improved quality of life for individuals with diabetes and musculoskeletal impairments.

## Data Availability

The datasets generated and/or analyzed during the current study are not publicly available to protect the privacy of the study participants but are available from the corresponding author upon reasonable request.

## References

[1] Larsson L, Degens H, Li M, Salviati L, Lee YI, Thompson W, Kirkland JL, Sandri M (2019). Sarcopenia: aging-related loss of muscle Mass and function. Physiol Rev.

[2] Wei S, Nguyen TT, Zhang Y, Ryu D, Gariani K (2023). Sarcopenic obesity: epidemiology, pathophysiology, cardiovascular disease, mortality, and management. Front Endocrinol.

[3] Cruz-Jentoft AJ, Bahat G, Bauer J et al. Sarcopenia: revised European consensus on definition and diagnosis [published correction appears in Age Aging. 2019;48(4):601]. *Age Aging*. 2019;48(1):16–31. 10.1093/aging/afy169 10.1093/ageing/afz046 PMC6593317 31081853

[4] Petermann-Rocha F, Balntzi V, Gray SR, Lara J, Ho FK, Pell JP, Celis-Morales C (2022). Global prevalence of Sarcopenia and severe Sarcopenia: a systematic review and meta-analysis. J cachexia Sarcopenia Muscle.

[5] Chen H, Huang X, Dong M, Wen S, Zhou L, Yuan X (2023). The Association between Sarcopenia and Diabetes: from pathophysiology mechanism to therapeutic strategy. Diabetes Metabolic Syndrome Obesity: Targets Therapy.

[6] Mesinovic J, Zengin A, De Courten B, Ebeling PR, Scott D (2019). Sarcopenia and type 2 diabetes mellitus: a bidirectional relationship. Diabetes Metabolic Syndrome Obesity: Targets Therapy.

[7] Purnamasari D, Tetrasiwi EN, Kartiko GJ, Astrella C, Husam K, Laksmi PW (2022). Sarcopenia and Chronic complications of type 2 diabetes Mellitus. Rev Diabet Studies: RDS.

[8] Donini LM, Busetto L, Bischoff SC, Cederholm T, Ballesteros-Pomar MD, Batsis JA, Barazzoni R (2022). Definition and diagnostic criteria for sarcopenic obesity: ESPEN and EASO consensus statement. Obes Facts.

[9] Gao Q, Mei F, Shang Y, Hu K, Chen F, Zhao L, Ma B (2021). Global prevalence of sarcopenic obesity in older adults: a systematic review and meta-analysis. Clin Nutr.

[10] Pal R, Bhadada SK, Aggarwal A, Singh T (2021). The prevalence of sarcopenic obesity in community-dwelling healthy Indian adults - the sarcopenic obesity-Chandigarh Urban Bone Epidemiological Study (SO-CUBES). Osteoporos Sarcopenia.

[11] Sravya SL, Swain J, Sahoo AK, Mangaraj S, Kanwar J, Jadhao P, Das S (2022). Sarcopenia in type 2 diabetes Mellitus: study of the modifiable risk factors involved. J Clin Med.

[12] Khadra D, Itani L, Tannir H, Kreidieh D, Masri DE, Ghoch ME (2019). Association between sarcopenic obesity and higher risk of type 2 diabetes in adults: a systematic review and meta-analysis. World J Diabetes.

[13] Salom Vendrell C, García Tercero E, Moro Hernández JB, Abel B (2022). Sarcopenia as a little-recognized comorbidity of type II diabetes Mellitus: a review of the diagnosis and treatment. Nutrients.

[14] Zhou Y, Wang J, Yao Q, Jian Q, Luo Z (2023). Prevalence of sarcopenic obesity in patients with diabetes and adverse outcomes: a systematic review and meta-analysis. Clin Nutr ESPEN.

[15] Riuzzi F, Sorci G, Arcuri C, Giambanco I, Bellezza I, Minelli A, Donato R (2018). Cellular and molecular mechanisms of Sarcopenia: the S100B perspective. J Cachexia Sarcopenia Muscle.

[16] Wu J, Ding P, Wu H, Yang P, Guo H, Tian Y, Meng L, Zhao Q (2023). Sarcopenia: molecular regulatory network for loss of muscle mass and function. Front Nutr.

[17] Narici MV, Maffulli N (2010). Sarcopenia: characteristics, mechanisms and functional significance. Br Med Bull.

[18] Maliszewska K, Adamska-Patruno E, Krętowski A (2019). The interplay between muscle mass decline, obesity, and type 2 diabetes. Pol Archives Intern Med.

[19] Chasapi A, Balampanis K, Kourea E, Kalfarentzos F, Lambadiari V, Lambrou GI, Melachrinou M, Sotiropoulou-Bonikou G (2018). Can obesity-induced inflammation in skeletal muscle and intramuscular adipose tissue accurately detect liver fibrosis?. J Musculoskel Neuronal Interact.

[20] Sinha I, Sakthivel D, Varon DE (2017). Systemic regulators of skeletal muscle regeneration in obesity. Front Endocrinol.

[21] Hildebrandt X, Ibrahim M, Peltzer N (2023). Cell death and inflammation during obesity: know my methods, WAT(son). Cell Death Differ.

[22] Beaudart C, Reginster JY, Petermans J, Gillain S, Quabron A, Locquet M, Slomian J, Buckinx F, Bruyère O (2015). Quality of life and physical components linked to Sarcopenia: the SarcoPhAge study. Exp Gerontol.

[23] Morley JE, Abbatecola AM, Argiles JM, Baracos V, Bauer J, Bhasin S, Cederholm T, Coats AJ, Cummings SR, Evans WJ, Fearon K, Ferrucci L, Fielding RA, Guralnik JM, Harris TB, Inui A, Kalantar-Zadeh K, Kirwan BA, Mantovani G, Anker SD (2011). Sarcopenia with limited mobility: an international consensus. J Am Med Dir Assoc.

[24] World Health Organization. Global Physical Activity Questionnaire (GPAQ). https://www.who.int/publications/m/item/global-physical-activity-questionnaire. Accessed 6/02/2024.

[25] Heymsfield SB, Lohman TG, Wang Z, Going SB, editors. Human body composition. Human Kinetics. 2005.

[26] Chen LK, Liu LK, Woo J, Assantachai P, Auyeung TW, Bahyah KS, Chou MY, Chen LY, Hsu PS, Krairit O, Lee JSW, Lee WJ, Lee Y, Liang CK, Limpawattana P, Lin CS, Peng LN, Satake S, Suzuki T, Suriyawongpaisal P (2014). Sarcopenia in Asia: Consensus report of the Asian Working Group for Sarcopenia. J Am Med Dir Assoc.

[27] Expert Panel on Detection, Evaluation, and Treatment of High Blood Cholesterol in Adults (2001). Executive summary of the third report of the National Cholesterol Education Program (NCEP) Expert Panel on detection, evaluation, and treatment of high blood cholesterol in adults (Adult Treatment Panel III). JAMA.

[28] Kim TN, Yang SJ, Yoo HJ (2009). Prevalence of Sarcopenia and sarcopenic obesity in Korean adults: the Korean sarcopenic obesity study. Int J Obes (Lond).

[29] Curtis M, Swan L, Fox R, Warters A, O’Sullivan M (2023). Associations between Body Mass Index and probable Sarcopenia in Community-Dwelling older adults. Nutrients.

[30] Yogesh M, Mody M, Makwana N, Rabadiya S, Patel J, Shah S (2024). The hidden battle within: shedding light on the coexistence of Sarcopenia and sarcopenic obesity among participants with type 2 diabetes in a tertiary care hospital, Gujarat. Indian J Endocrinol Metabol.

[31] Gregori G, Paudyal A, Barnouin Y, Celli A, Segoviano-Escobar MB, Armamento-Villareal R, Napoli N, Qualls C, Villareal DT (2023). Indices of sarcopenic obesity are important predictors of finite element analysis-derived bone strength in older adults with obesity. Front Endocrinol.

[32] Wang M, Tan Y, Shi Y, Wang X, Liao Z, Wei P (2020). Diabetes and sarcopenic obesity: Pathogenesis, diagnosis, and treatments. Front Endocrinol.

[33] He Q, Wang X, Yang C, Zhuang X, Yue Y, Jing H, Hu J, Sun M, Guo L. Metabolic and nutritional characteristics in Middle-aged and Elderly Sarcopenia patients with type 2 diabetes. J Diabetes Res. 2020;2020(6973469). 10.1155/2020/6973469 10.1155/2020/6973469 PMC7661113 33204732

[34] Lisco G, Disoteo OE, De Tullio A, De Geronimo V, Giagulli VA, Monzani F, Jirillo E, Cozzi R, Guastamacchia E, De Pergola G, Triggiani V (2023). Sarcopenia and Diabetes: a detrimental Liaison of advancing age. Nutrients.

[35] Seo JH, Lee Y. Association of physical activity with Sarcopenia evaluated based on muscle mass and strength in older adults: 2008–2011 and 2014 – 2018 Korea national health and nutrition examination surveys. BMC Geriatr. 2022;22(1). 10.1186/s12877-022-02900-3 10.1186/s12877-022-02900-3 PMC8928682 35296249

[36] Habib SS, Alkahtani S, Alhussain M, Aljuhani O (2020). Sarcopenia coexisting with high adiposity exacerbates insulin resistance and Dyslipidemia in Saudi Adult men. Diabetes Metabolic Syndrome Obesity: Targets Therapy.

[37] Han P, Yu H, Ma Y, Kang L, Fu L, Jia L, Chen X, Yu X, Hou L, Wang L, Zhang W, Yin H, Niu K, Guo Q (2017). The increased risk of Sarcopenia in patients with cardiovascular risk factors in Suburb-Dwelling older Chinese using the AWGS definition. Sci Rep.

[38] Damluji, A. A., Alfaraidhy, M., Alhajri, N., Rohant, N., Kumar, M., Malouf, C. A.,… Goyal, P. (2023). Sarcopenia and cardiovascular diseases. Circulation, 147(20),1534–1553. https://doi.org/10.1161/circulationaha.123.064071. 10.1161/CIRCULATIONAHA.123.064071 PMC10180053 37186680

[39] Kim TN, Yang SJ, Yoo HJ, Lim KI, Kang HJ, Song W, Baik SH, Choi DS, Choi KM (2021). Sarcopenic obesity and insulin resistance according to the presence of metabolic syndrome. Endocrine.

[40] Hong SH, Choi KM (2020). Sarcopenic Obesity, insulin resistance, and their implications in Cardiovascular and metabolic consequences. Int J Mol Sci.

[41] Kreidieh D, Itani L, El Masri D, Tannir H, Citarella R, Ghoch E (2018). Association between Sarcopenic Obesity, type 2 diabetes, and hypertension in overweight and obese treatment-seeking adult women. J Cardiovasc Dev Disease.

[42] Volpato S (2014). Prevalence and clinical correlates of Sarcopenia in community-dwelling older people: application of the EWGSOP definition and diagnostic algorithm. J Gerontol Ser Biol Sci Med Sci.

[43] Fukuda T, Bouchi R, Takeuchi T, Tsujimoto K, Minami I, Yoshimoto T, Ogawa Y (2018). Sarcopenic obesity assessed using dual energy X-ray absorptiometry (DXA) can predict cardiovascular disease in patients with type 2 diabetes: a retrospective observational study. Cardiovasc Diabetol.

[44] Remelli F, Maietti E, Abete P, Bellelli G, Bo M, Cherubini A, Corica F, Di Bari M, Maggio M, Rizzo MR, Rossi AP, Landi F, Volpato S, GLISTEN Group Investigators (2022). Prevalence of obesity and diabetes in older people with Sarcopenia defined according to EWGSOP2 and FNHI criteria. Aging Clin Exp Res.

[45] Santos MD, Buti M, López-Cano C, Sánchez E, Vidal A, Hernández M, Lafarga A, Gutiérrez-Carrasquilla L, Rius F, Bueno M, Lecube A (2020). Dynamics of Anthropometric Indices in a large paired cohort with 10 years of Follow-Up: paving the way to sarcopenic obesity. Front Endocrinol.

[46] Merchant RA, Soong JTY, Morley JE (2022). Gender differences in body composition in Pre-frail older adults with diabetes Mellitus. Front Endocrinol.

